# Traumatic Meniscus and Cruciate Ligament Tears in Young Patients: A Comparison of 3T Versus 1.5T MRI

**DOI:** 10.5334/jbr-btr.1158

**Published:** 2017-03-29

**Authors:** Nasreddine Nouri, Mouna Chelli Bouaziz, Hend Riahi, Meriem Mechri, Abdelhakim Kherfani, Moez Ouertatani, Mohamed Fethi Ladeb

**Affiliations:** 1 Institut Kassab D’orthopedie; Ksar Said, TN

**Keywords:** Anterior cruciate ligament, Arthroscopy, Knee injury, Lateral meniscus, Magnetic resonance imaging, Medial meniscus

## Abstract

**Objective:**

To compare diagnosis value of 1.5T and 3T MRI in the detection of traumatic knee injuries in young patients by reference to arthroscopy.

**Materials and Methods:**

One hundred patients were prospectively included. All patients randomly underwent standardized knee 1.5T or 3T MRI with subsequent knee arthroscopy. Meniscus and cruciate ligaments tears were blindly assessed by two independent musculoskeletal radiologists.

**Results:**

Comparison of 1.5T and 3T MRI groups in the diagnosis of medial and lateral meniscal tears showed significantly higher sensitivity (*p* = 0.015) of 1.5T MRI in the diagnosis of lateral meniscal tears. Sensitivity and specificity for complete ACL tears were 100 percent [35/35] and 100 percent [23/23] at 1.5T MRI (*p* = < 0.0001) versus 95.5 percent [21/22] and 100 percent [16/16] at 3T MRI (*p* = < 0.0001).

Only three complete PCL tears were observed in this study. Sensitivity and specificity for all complete CL tears were 100 percent [37/37]; 100 percent [77/77] for 1.5T MRI (*p* < 0.0001); and 95.7 percent [22/23] and 100 percent [59/59] for 3-T MRI (*p* < 0.0001). Diagnosis value of 1.5T and 3T MRI was equal for ACL and PCL complete tears.

**Conclusion:**

Diagnosis value of 1.5T was similar to 3T MRI for medial meniscal and cruciate ligament tears of the knee in symptomatic patients and higher for lateral meniscal tears.

## Introduction

Magnetic resonance imaging (MRI) is a non-invasive diagnosis procedure and is considered the method of choice for assessing traumatic knee injuries. Most of the studies evaluating the diagnostic value of MRI in the assessment of knee abnormalities were performed with 1.5T MRI or lower field strength [1,2].

The use of high field strength 3T MRI systems is becoming widespread in clinical practice, and MRI equipment is shifting from 1.5T to 3T. Few studies comparing 1.5T versus 3T have been performed. Most of these showed no improved performance for 3T MRI [3,4,5,6], whereas experimental studies have shown improved diagnosis value of 3T compared with 1.5T MRI in visualization of anatomical structures [7,8,9,10].

The purpose of this prospective study is to compare the diagnosis value of 1.5T and 3T MRI in meniscal tears and cruciate ligaments lesions, using arthroscopy as a gold standard.

## Materials and Methods

This prospective study was performed with approval from our institutional review board.

- Study Group

One hundred consecutive symptomatic patients (86 men and 14 women; mean age 30 years +/– 8.22; range 16–52 years) were included who underwent MRI of the knee and subsequent arthroscopic knee surgery. Patients were randomly assessed by 3T MRI (*n* = 56) or by 1.5T MRI (*n* = 44).

- Inclusion and Non-Inclusion Criteria

Patients aged from 16 to 55 years who had experienced symptoms such as pain, swelling, instability, or locking after a knee trauma and who had been consecutively referred to the departments of orthopedics at our institution between January and October 2013 were included in this study. MRI protocol was standardized for all patients, and arthroscopy was performed within a maximum of 90 days after MRI. Patients with a history of knee surgery, severe osteoarthritis of the knee, and contraindications for MRI were not included in the study.

- MRI Examinations

All 100 patients in the study group underwent MRI examinations performed either with 1.5T (General Electric Healthcare Systems Signa HD) or 3T (Siemens Magnetom Verio) equipment using an eight-channel knee coil on each system. The decision on whether to evaluate a patient at 1.5T or 3T was made randomly. All patients were evaluated using the same imaging protocol (Table 1).

**Table 1:** Imaging Parameters of MR protocols.

	SAGITTAL PD		CORONAL PD		SAGITTALPD FS		CORONALPD FS		AXIALPD FS 4 mm		AXIALPD FS 2 mm		AXIAL OBLIQUE PD	
	
	3T	1.5T	3T	1.5T	3T	1.5T	3T	1.5T	3T	1.5T	3T	1.5T	3T	1.5T

**Repetition time (ms)**	3000	1640	3000	1700	2040	3060	1740	2020	2080	2040	2080	2040	3000	1640
**Echo time(ms)**	36	35	36	33.7	33.66	34.3	33.66	33.2	34.34	33.5	34	33.5	36	48
**Field of view (cm)**	18 × 18	16 × 16	18 × 18	18 × 18	18 × 18	16 × 16	18 × 18	18 × 18	19 × 19	19 × 19	16 × 16	19 × 19	20 × 18	16 × 16
**Section thickness (mm)**	3	4	3	4	4	4	4	4	4	4	2	2	2	2
**INTERVAL**	0.5	1	0.5	1	0.5	1	0.5	1	0.5	1	0.2	0.2	0.2	0.2
**Matrix size**	384 × 312	320 × 256	384 × 312	320 × 256	320 × 256	320 × 256	320 × 256	320 × 256	320 × 256	320 × 256	352 × 256	352 × 256	256 × 256	256 × 256
**Number of slices**	24	20	24	20	24	20	24	20	24	24	24	24	24	24
**Imaging time**	3′40	2′24	3′50	2′00	3′30	2′24	3′35	2′22	3′10	2′23	2′35	2′35	3′40	2′15

- MRI Results

All MRI examinations were interpreted by one of two academic musculoskeletal radiologists with 12 and 20 years’ experience in MRI musculoskeletal imaging. The radiologists were not aware of clinical findings before MRI.

Meniscal tears were diagnosed in the presence of abnormal morphologic features of the meniscus or abnormal meniscal signal reaching an articular surface on one or more MRI images. If a meniscal tear was diagnosed, the reader recorded the tear location at the anterior horn, body, or the posterior horn of the meniscus. Meniscal tears were classified as complex, unstable, horizontal, radial, or peripheral longitudinal [11,12,13].

All radiologists and orthopedic surgeons agreed to catalogue both flap tears and bucket handle tears as unstable tears. The MR imaging signs of an unstable tear included any of the following: notch fragment, presence of recess fragments, double posterior cruciate ligament sign, flipped-meniscus sign, absence of the bow-tie sign, too-tall anterior horn sign, and disproportionate posterior horn sign [11,14].

Anterior (ACL) and posterior (PCL) cruciate ligaments abnormalities were assessed as complete or partial tears. A cruciate ligament (ACL or PCL) was considered as completely torn when any of the following primary signs were identified: partial or total discontinuity in at least one reading plane [2,15]; horizontalisation of the distal portion (ACL) [2]; a focal or diffuse high signal within the ligament; irregular, fuzzy, ill-defined contours with thickening of the ligament; or complete lack of visualization of the ligament [15].

- Arthroscopic Findings

Arthroscopic knee surgery was performed in all patients within three weeks (range 1–13 weeks; mean 3 weeks) of MRI examination. Orthopedic surgeons were aware of the MRI findings of all patients at the time of arthroscopy. All arthroscopic procedures were performed at the same institution by one of seven fellowship-trained academic orthopedic surgeons with experience in knee arthroscopy. Video recording was performed during arthroscopy. The orthopedic surgeon was asked to assess the location of meniscal tears in the anterior horn, body, or the posterior horn of the meniscus and to classify them into types as complex, unstable, horizontal, radial, or peripheral longitudinal [11,16].

A complete ACL tear is diagnosed arthroscopically if the ligament is absent in the intercondylar notch region or if there is loss of ligament continuity with only ligament remnants at each end. In cases where continuity of one or both bundles of the ligament was present and laxity of the ligament was noted by probing, the injury was defined as a partial lesion. Arthroscopic signs of complete PCL tears were the same as ACL tears.

## Statistical Analysis

Two-by-two contingency tables were used to calculate sensitivities and specificities, positive and negative predictive values for the medial and lateral menisci, and ACL and PCL lesions for MRI in both 1.5T and 3T groups, with the arthroscopic findings as the gold standard for the presence or absence of a lesion as well as location and configuration of meniscal tears. Statistical significance was defined as *p* < 0.05.

The Epitable of Epi Info 6 program (CDC, Atlanta Georgia (USA) 2001) was used to calculate confidence interval and to compare the sensitivities and specificities in 1.5T and 3T groups.

## Results

**Figure 1:**
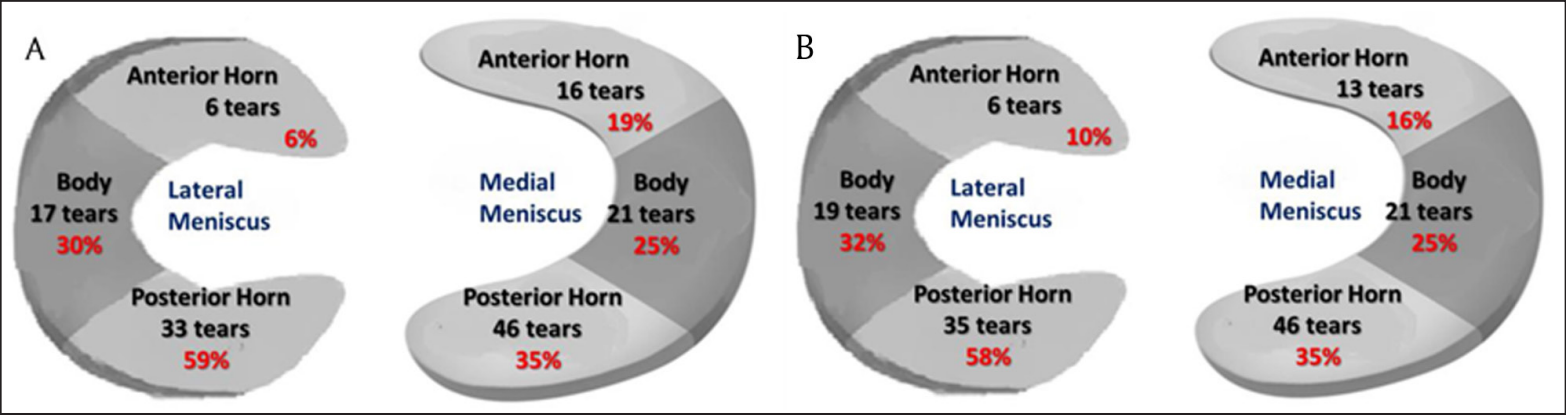
**(a)** Meniscal tear distribution on MRI **(b)** Meniscal tear distribution at arthroscopy.

**Figure 2:**
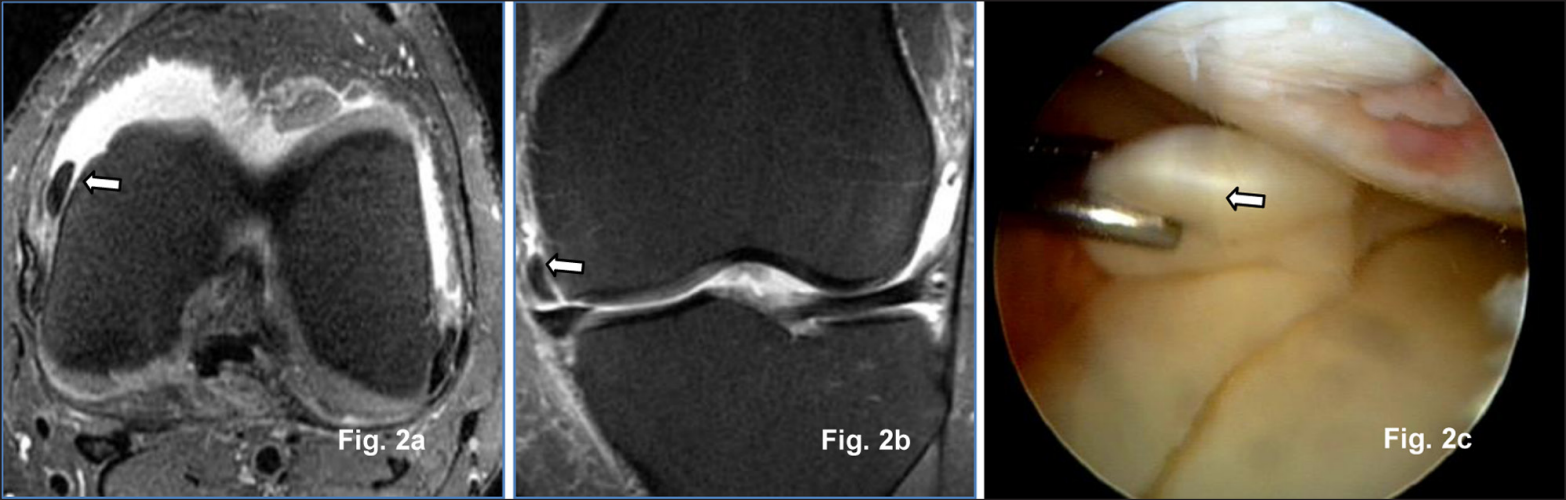
Transversal **(a)** and coronal PD Fat Sat **(b)** 1.5-T MR images: Medial meniscus unstable lesion in medial para-condylar recess (arrow) confirmed at arthroscopy (arrow) **(c).**

**Figure 3:**
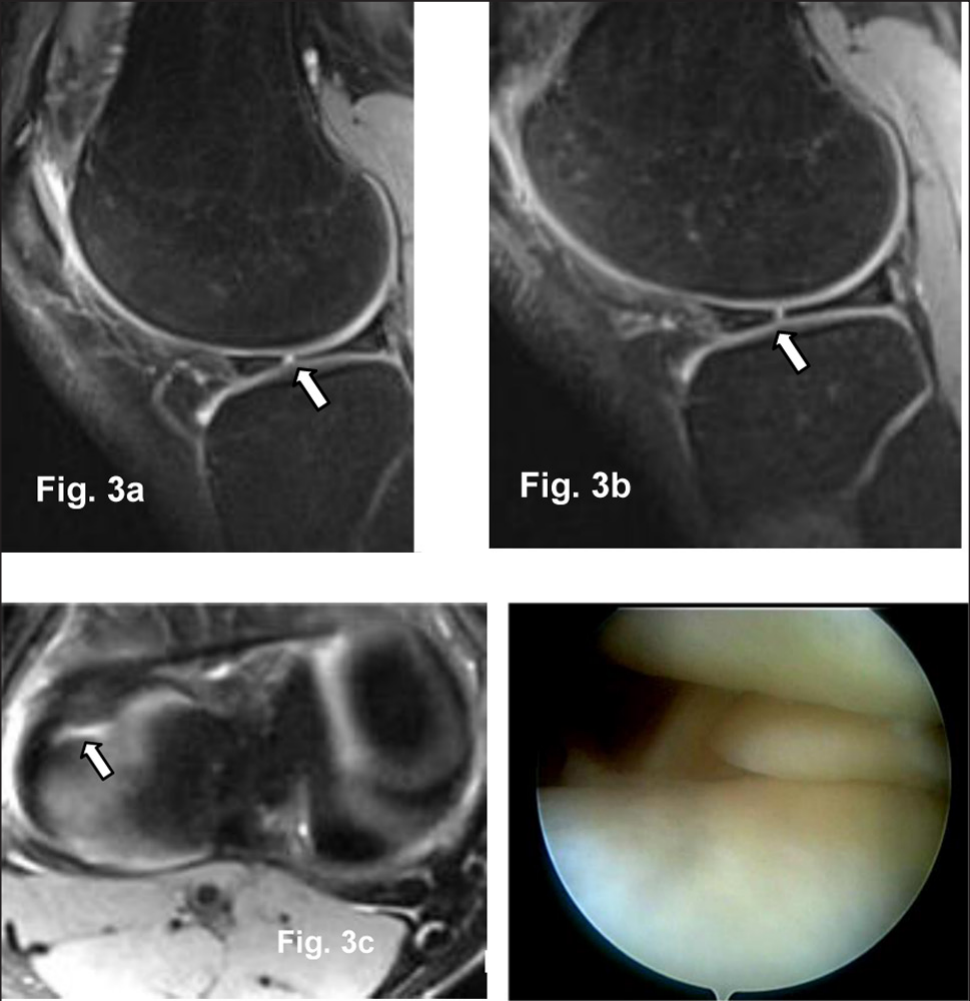
Sagittal **(a, b)** and transversal 2 mm PD Fat Sat 3T MR images **(c)**: Radial tear in the body of lateral meniscus (arrow) confirmed at arthroscopy **(d).**

➢ Meniscal tears (Figures 1, 2, and 3)

Fifty-three medial and 46 lateral meniscal tears were arthroscopically identified in all 100 patients. MRI sensitivity, specificity, positive and negative predictive values for medial meniscus, lateral meniscus, and both menisci tears are summarized in Table 2.

**Table 2:** Diagnosis Value of MRI in Meniscal Tears.

	Medial meniscus			Lateral meniscus			All meniscal tears		
	
	MRI	1.5T	3T	MRI	1.5T	3T	MRI	1.5T	3T

**True positive**	49	30	19	39	20	19	88	50	38
**True negative**	45	22	23	57	35	22	120	75	45
**False positive**	2	1	1	1	1	0	3	2	1
**False negative**	4	3	1	3	0	3	7	3	4
**Sensitivity**	92.5%	90.90% [74.5–97.6]	95.0% [73.1–99.7]	89.1%	100% [80–100]	86.4% [64–96.4]	92.6%	94% [80–100]	90.5% [80–100]
**Confidence interval**	0.64			0.015			0.88		
**Specificity**	95.7%	95.7% [76–99.8]	95.8% [76.9–99.8]	98.3%	97.2% [83.8–99.9]	100.0% [81.5–100]	96.7%	96.6% [81.5–100]	97.8% [81.5–100]
**Confidence interval**	0.78			0.81			0.92		
**Positive predictive value**	96.1%	96.80%	95.0%	97.5%	95.5%	100.0%	96.7%	96.15%	97.4%
**Negative predective value**	91.8%	88.0%	95.8%	91.9%	94.6%	88.0%	92.9%	95%	91.8%
***p***	< 0.0001	< 0.001	< 0.001	< 0.001	< 0.001	< 0.0001	< 0.0001	< 0.0001	< 0.0001

A comparison of the 1.5T and 3T MRI groups shows significantly higher sensitivity (*p* = 0.015) of 1.5T MRI in the diagnosis of lateral meniscal tears (Table 2). Specificity for lateral meniscus (*p* = 0.81), sensitivity (*p* = 0.64), and specificity (*p* = 0.78) for medial meniscus were not significantly different for 1.5T versus 3T.

✓ Meniscal tear configuration

A comparison of 1.5T and 3T MRI in the detection of tear configuration is summarized in Table 3. The 3T MRI showed a significantly higher sensitivity in the assessment of horizontal (*p* < 0.0001) and unstable tears (*p* = 0.005) in medial meniscus compared to 1.5T MRI. The diagnosis value of complex tears of the medial meniscus was similar for both groups (sensitivity *p* = 1; specificity *p* = 0.83). Because of the small number of longitudinal, oblique, radial, and horizontal tears in the lateral meniscus, no statistical comparison was obtained.

**Table 3:** Comparison of 1.5-T and 3-T MRI Diagnosis Value in Meniscal Tears Configuration.

	Configuration of medial meniscus tear						Configuration of lateral meniscus tear			
	
	Horizontal		Complex		Unstable		Complex		Unstable	
	
	1.5 T	3T	1.5 T	3T	1.5 T	3T	1.5 T	3 T	1.5T	3T

**True positive**	4	3	8	5	8	8	6	8	5	7
**True negative**	46	38	46	38	46	36	48	34	49	35
**False positive**	3	3	2	1	0	0	2	0	0	0
**False negative**	3	0	0	0	2	0	0	2	2	2
**Sensitivity**	57.10% [20.2–88.2]	100.00% [31–100]	100.00% [59.8–100]	100.00% [46.3–100]	80.00% [44.2–96.5]	100.00% [59.8–100]	100.00% [59.8–100]	100.00% [46.3–100]	80.00% [44.2–96.5]	100.00% [59.8–100]
**Confidence interval**	<0.0001		1		0.005		1		0.005	
**Specificity**	93.90% [82.1–98.4]	92.70% [79–98.1]	95.80% [84.6–99.3]	97.40% [84.9–99.9]	100.00% [90.4–100]	100.00% [88–100]	95.80% [84.6–99.3]	97.40% [84.9–99.9]	100.00% [90.4–100]	100.00% [88–100]
**Confidence interval**	0.9		0.83		1		0.83		1	

The 3T MRIs showed statistically higher sensitivity in the diagnosis of lateral meniscus unstable tears (*p* = 0.005), whereas no statistical difference was noted in the specificity (*p* = 1). Diagnosis value in the detection of lateral meniscus complex tears was similar in both groups (sensitivity *p* = 1; specificity *p* = 0.83).

✓ Meniscal tear location

A comparison of the 1.5T and 3T MRI groups in the assessment of tear locations is summarized in Table 4. The diagnosis value of 3T and 1.5T MRI was similar in the detection of the location of medial meniscus tears. Given the paucity of tears in the anterior horn of the lateral meniscus, no statistical results could be obtained for those tears. A 1.5T MRI showed statistically higher sensitivity in the diagnosis of tear locations in the body (*p* = 0.006) and posterior horn (*p* = 0.0006) of the lateral meniscus compared to 3T MRI, whereas no statistical difference was noted in the specificity.

**Table 4:** Comparison of 1.5-T and 3-T MRI in the Assessment of Meniscal Tears Location.

	Location of medial meniscus tear						Location of lateral meniscus tear			
	
	Anterior horn		Body		Posterior horn		Body		Posterior horn	
	
	1.5 T	3T	1.5 T	3T	1.5-T	3T	1.5-T	3T	1.5-T	3T

**True positive**	9	3	11	7	26	13	7	10	17	14
**True negative**	46	37	43	33	25	25	49	32	38	25
**False positive**	0	4	1	2	2	2	0	0	1	1
**False negative**	1	0	1	2	3	4	0	2	0	4
**Sensitivity**	90.00% [54.1–99.5]	100.00% [31–100]	91.70% [59.8–99.6]	77.80% [40.2–96.1]	89.70% [71.5–97.3]	76.50% [49.8–92.2]	100.00% [56.1–100]	83.30% [20.9–97.1]	100.00% [77.1–100]	77.80% [51.9–92.6]
**Confidence interval**	0.069		0.055		0.1		0.006		0.0006	
**Specificity**	100.00% [90.4–100]	90.20% [75.9–96.8]	97.70% [86.5–99.9]	94.30% [79.5–99]	92.60% [74.2–98.7]	92.60% [74.2–98.7]	100.00% [90.1–100]	100.00% [86.7–100]	97.40% [84.9–99.9]	96.20% [78.4–99.8]
**Confidence interval**	0.07		0.44		0.74		1		0.83

**Figure 4:**
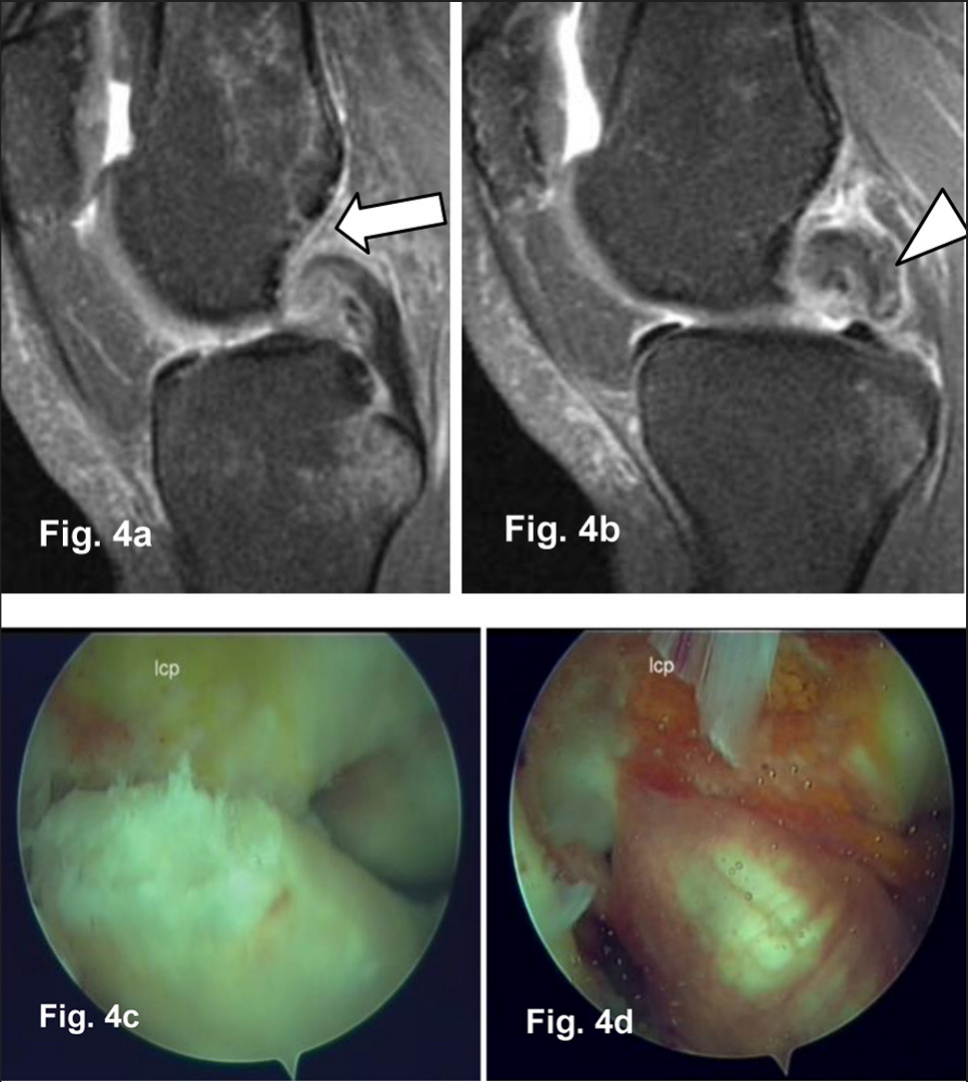
Sagittal PD Fat Sat 1.5T MR Images **(a, b)**: Complete ACL (arrow) and PCL (arrowhead) tear confirmed by arthroscopy **(c, d).**

- ➢ Cruciate ligament tears (Figure 4)

Sixty-two ACL and three PCL tears were identified by arthroscopy. All PCL tears were complete, whereas ACL tears were complete in 57 patients and partial in 5 patients. The diagnosis value of 3T and 1.5T MRI were similar in the detection of complete ACL and PCL lesions.

Table 5 summarizes sensitivity, specificity, and positive and negative predictive value for cruciate ligaments tears for 1.5T and 3T. Because of the small number of partial ACL tears, no statistical comparison could be obtained for these lesions.

**Table 5:** MRI Diagnosis Value in the Assessment of Cruciate Ligament Tears.

	ACL complete tears			PCL complete tears			CL complete tears		
	
	MRI	1.5T	3T	MRI	1.5T	3T	MRI	1.5 T	3T

**True positive**	56	35	21	3	2	1	59	37	22
**True negative**	39	23	16	97	54	43	136	77	59
**False positive**	0	0	0	0	0	0	0	0	0
**False negative**	1	0	1	0	0	0	1	0	1
**Sensitivity (Confidence Interval)**	98.2% [89,4–99,9]	100.0% [87,7–100]	95.5% [75,1–99,8]	100% [31,0–100]	100.00% [19,8–100]	100% [5,5–100]	98.3% [89,9–99,9]	100% [88,3–100]	95.7% [76–96,8]
**Specificity (confidence interval)**	100% [88,8–100]	100.0% [82.2–100]	100% [75,9–100]	100% [95,3–100]	100% [91,7–100]	100% [89,8–100]	100% [96,6–100]	100% [94,1–100]	100% [92,4–100]
**Positive predictive value**	100% [92,0–100]	100.0% [87,7–100]	100% [80,8–100]	100% [31,0–100]	100% [19,8–100]	100% [5,5–100]	100% [92,4–100]	100% [88, 3–100]	100% [81,5–100]
**Negative predective value**	97.5% [85,3–99,9]	100.00% [82.2–100]	94,1% [69,2–99,7]	100% [95,3–100]	100% [91,7–100]	100% [89,8–100]	99.3% [95,4–100]	100% [94,1–100]	98.3% [89,9–99,9]
***p***	< 0.0001	< 0.0001	< 0.0001	< 0.001	0.01	0.023	< 0.0001	< 0.0001	< 0.0001

## Discussion

Our study showed that 3T MRI of the knee does not improve diagnosis accuracy compared to 1.5T MRI for detecting meniscal and cruciate ligaments lesions. We found no significant difference between 3T MRI and 1.5T MRI groups in the diagnosis of medial meniscal tears. We found significantly increased sensitivity in assessing lateral meniscal abnormalities at 1.5T.

In the assessment of meniscal tear types, our study showed that 3T overperforms 1.5T to detect horizontal and unstable tears for medial meniscus and that 1.5T is more reliable than 3T for unstable tears of the lateral meniscus.

Few previous reports have compared knee MRI at 1.5 and 3T in the detection of meniscal tears and central pivot lesions. However, these studies were performed in different patient populations or included only a small number of patients. A review of articles published between 1991 and 2000 revealed no difference in the ability to detect meniscal tears with MRI units with magnets varying in strength from 0.1 to 1.5T [1]. Few studies have compared 1.5 and 3T MRI with reference to the arthroscopy.

These studies do not all conclude that 3T MRI is superior to 1.5T MRI. In a recent prospective study, Van Dyck et al. [6] showed no significant improvement of 3T MRI diagnosis accuracy either for meniscal or anterior cruciate ligament lesions compared to 1.5T MRI. Magee et al. [17] and Ramanth et al. [18] suggested superiority of 3T MRI by comparing their own results to the results of previous studies using 1.5T or lower fields. But in these studies, no direct comparison with imaging at 1.5 T was available, and the results were compared with previously published results.

In a retrospective study, Grossman et al. [3] compared 100 consecutive patients who underwent 3T MRI of the knee and 100 consecutive patients who underwent 1.5T MRI of the knee to determine the accuracy of MRI in meniscal tear diagnosis knee arthroscopy as the reference standard. In this study, the authors concluded at a similar value of 1.5T and 3T MRI for diagnosis of meniscal tears. In the same study, the causes of false positives and false negatives for meniscal tears on MRI were similar for 1.5 and 3T MRI field strength.

Similarly, Krampla et al. [4] found no superiority of 3T MRI compared to 1.5T and 1T. In this study, the inter-observer reliability in the interpretation of meniscal tears, degree of chondropathy, and ACL integrity were analyzed while taking the radiologist’s experience and field strength into account. The authors considered that the radiologist’s experience was more discriminating than the magnetic field strength for MRI diagnosis value.

Schoth et al. [19] compared knee anatomic structures visualization and found superior subjective visualization at 3T versus 1.5T for menisci and ligaments; however, no dedicated evaluation of meniscal tears with arthroscopic correlation was available in this study.

Few studies have compared the diagnosis value of 1.5T and 3T MRI in assessing ACL lesions. A review of articles published between 1991 and 2000 [1] suggests that higher magnetic field strength significantly increases diagnosis MRI value in assessing anterior cruciate ligament lesions. To the best of our knowledge, no previous study has compared the value of 1.5T and 3T knee MRI for assessing PCL lesions using arthroscopy as a reference standard. Our results show that 3T and 1.5T MRI have similar results in the detection of complete PCL lesions.

The prospective design, the breakdown of meniscal and cruciate ligaments tears by type and location, and the correlation with arthroscopic findings constitute the strength of this study. The main study limitations are the use of MRI machines of two different manufacturers and lack of inter- and intra-observer variability assessment. Moreover, seven different surgeons leaving possible errors of judgment depending on the surgeon’s experience performed arthroscopy.

Our study population was young, with a mean age of 30 years. Our results may not be extended to the general population, but they may apply to other patients with similar conditions in similar institutions.

In our study, the images are thicker at 1.5T than at 3T (3 mm vs. 4 mm). Slice thickness is responsible for the spatial resolution. Using thin slices improves the spatial resolution and less partial volume artifact. We used a slice thickness of 4 mm in 1.5T to compensate the decrease of the signal. However, sensitivity of the sequence is mostly related to contrast parameters such as repetition time (TR), echo time (TE), and flip angle (FA). So there was no big change in contrast when working with 3 mm or 4 mm.

The assessment of two different cohorts on 1.5T and 3T MRI is not a real limitation because patients were randomly distributed.

## Conclusion

Our study shows that 1.5T MRI has improved diagnosis value for evaluating lateral meniscal tears; meanwhile, for some tears, such as medial meniscus horizontal and unstable tears, 3T is better than 1.5T. The diagnosis value of 1.5T MRI was similar for cruciate ligament complete tears of the knee joint in symptomatic patients when compared to 3T MRI.
